# A comparison of the effect of xinruibai versus filgrastim on hematopoietic reconstruction after allogeneic hematopoietic stem cell transplantation

**DOI:** 10.1186/s13052-018-0482-0

**Published:** 2018-05-31

**Authors:** Qixiang Ye, Hebi Jiang, Hua Jiang

**Affiliations:** Department of Hematology and Oncology, Guangzhou Women and Children’s Medical Center, No.9 Jinsui Road, Guangzhou, 510623 Guangdong Province China

**Keywords:** Pegfilgrastim, Filgrastim, Hematopoietic stem cell transplantation, Hematopoietic reconstruction

## Abstract

**Background:**

To compare the effect of xinruibai (Pegfilgrastim) and filgrastim injections on white blood cell and platelet (PLT) recovery, adverse events, post-operative complications, and cost effectiveness after allogeneic hematopoietic stem cell transplantation (allo-HSCT).

**Methods:**

Children who underwent allo-HSCT at our hospital from January 2014 to May 2017 due to thalassemia major, aplastic anemia, leukemia, and mucopolysaccharidosis were included. Among the children, 53 received xinruibai injections and 33 received filgrastim injections.

**Results:**

There were no significant differences in the average time to neutrophil and platelet recovery, the incidence of post-operative complications after allo-HSCT, the number of red blood cell and PLT infusions, or the incidence of adverse events related to the injection between two groups (*P* >  0.05). The pain score was 3.06 (SD 0.41) for the xinruibai group and 25.18 (SD 6.22) for the filgrastim group, indicating significant differences between the two groups (*P* <  0.001). No difference was found in the hospitalization cost. The cost of the granulocyte-colony stimulating factor (G-CSF) was 257.11 ± 61.87 Euro in the xinruibai group and 214.79 ± 0.00 Euro in the filgrastim group, showing significant difference (*P* <  0.001).

**Conclusions:**

Xinruibai injection was more convenient, simple, effective, and safer than filgrastim.

## Background

Allogeneic hematopoietic stem cell transplantation (allo-HSCT) is an important treatment for hematologic malignancies, metabolic disease, and immunologic deficiency. Granulocyte-colony stimulating factor (G-CSF) infusion can promote hematopoiesis, accelerate reconstruction of the immune system, reduce the duration of febrile neutropenia (FN), decrease the incidence of infections and the number of infusions of blood products, and shorten the hospitalization stay [1–6]. Pegfilgrastim and filgrastim are two common G-CSF injections. Compare with filgrastim,the N-terminal amino acid of pegfilgrastim is linked to a 20 k Dalton, which increases its half-life and reduces the likelihood of being degraded. The clearance of pegfilgrastim is mainly mediated by the rhG-CSF receptor on the surface of neutrophils. As the absolute neutrophil count (ANC) in peripheral blood increases, the plasma level of PEG-rhG-CSF decreases, which gives rise to a self-regulation effect [7, 8]. It has been shown that pegfilgrastim is comparable to filgrastim in its ability to recruit autologous stem cells for transplantation after chemotherapy for solid tumors and to promote restoration of the immune system and hematopoietic reconstruction [9–13]. In addition, pegfilgrastim has the extra benefits of needing a smaller number of infusions, higher cost effectiveness, and convenient administration. Because of these features, pegfilgrastim is recommended for use by the American Society of Clinical Oncology (ASCO) and the European Organization for Research and Treatment of Cancer (EORTC) [14, 15]; however, very few clinical data are available regarding the use of pegfilgrastim after allo-HSCT for non-solid tumors in children. This study was a retrospective analysis comparing pegfilgrastim and filgrastim after allo-HSCT for non-solid tumors in children.

## Methods

### Patients

A retrospective study design was adopted. Children who received allo-HSCT at our hospital from January 2014 to May 2017 due to thalassemia major, aplastic anemia, leukemia, and mucopolysaccharidosis were included. Pre-treatment was divided into myeloablative and non-myeloablative regimens, and the myeloablative regimen consists of chemotherapy with or without radiotherapy. Among the children, 53 received xinruibai injections and 33 received filgrastim injections randomly. All of the patients underwent primary HSCT. Before the transplantation, the absolute neutrophil count (ANC) was ≥1.5 G/L and the platelet (PLT) count was ≥100 G/L on routine blood testing. The patients received an infusion of CD34^+^ cells at a dose ≥2.0 × 10^6^. Informed consent was obtained regarding the risk of HSCT and the use of G-CSF.

#### G-CSF treatment procedures

xinruibai (xinruibai) was manufactured by Qilu Pharmaceutical (3 mg/bottle; China) and highly consistent with PEG-G-CSF (neulastim) manufactured by Amgen (Roche Scientific Company,USA). Filgrastim (75 μg/bottle; Kyowa Hakko Kirin Co., Ltd., China) was manufactured using a DNA recombinant technique.

Xinruibai or filgrastim was injected subcutaneously 5 days after HSCT. The dose of a single injection was 100 μg/kg in the xinruibai group and 5 μg/kg/d in the filgrastim group until the neutrophil count was ≥1.0 G/L for 3 consecutive days or a single neutrophil count ≥10.0 G/L plus a PLT count ≥20.0 G/L for 7 consecutive days. This was also the criterion for hematopoietic reconstruction. All patients were followed for at least 3 months. The patients later underwent sex chromosome determination using the FISH technique and/or microsatellite detection using STR-PCR during the course of follow-up to determine the state of the post-transplantation donor cells.

#### Management and endpoints

The patients underwent daily routine blood testing (twice weekly after achieving hematopoietic recovery), liver and kidney function testing twice weekly, and quantitative DNA analysis for cytomegalovirus and Epstein-Barr virus after transplantation. Hematopoietic recovery and transplantation-related complications, such as febrile neutropenia (FN), graft-versus-host disease (GVHD), and hemorrhagic cystitis, were observed. FN was defined as a fever > 38.4 °C once or > 38.2 °C on 3 consecutive readings with an ANC < 0.5 G/L [16]. The incidence of adverse events (AEs) of G-CSF was compared between the two groups (grade P3 AEs related or unrelated to the study drugs were assessed using NCI-CTCAE [version 3.0]), along with the pain index(A score of 0 indicated no pain, a score of 1–3 indicated mild pain, a score of 4–6 indicated moderate pain, and a score of 7–10 indicated severe pain), cost of G-CSF, and hospitalization cost (total cost from pre-treatment to discharge). Other routine therapies included infusion of blood products. Packed red blood cell and PLT transfusions were administered when hemoglobin (Hb) or PLT levels were < 8 g/dL or < 20.0 G/L, respectively. All blood products were irradiated and filtered prior to infusion [17]. Antibiotics and antiviral drugs were given depending on the conditions of the patient. During this study period the guidelines for supportive therapies adopted by the transplant team did not vary.

### Statistical analyses

All statistical analyses were performed using SPSS 19.0 software. Date obeying a normal distribution or quasi-normal distribution were described by ±s. Data not obeying a normal distribution were described by M(QR). Intergroup comparisons of means, medians, and categorical variables were performed using t-tests, Mann–Whitney U tests, and χ^2^ tests, respectively.

## Result

### Baseline clinical features

The two groups of patients had no significant differences in constitution, age, gender, body weight, pre-treatment, and number of infused CD34^+^ cells **(**Table 1**)**.

**Table 1:** Baseline clinical features of patients

Variable	xinruibai group (*N* = 53)	Filgrastim group (*N* = 33)	t/χ^2^ value	*P* value
Age	6.26 ± 3.05	7.30 ± 3.27	1.46	0.1467
Gender n (%)			0.16	0.681
Male	35 (66.0)	24 (72.7)		
Female	18 (34.0)	9 (27.3)		
Disease n (%)			2.54	0.198
Aplastic anemia	2 (3.8)	3 (9.1)		
Leukemia	2 (3.8)	6 (18.1)		
Thalassemia major	46 (86.8)	24 (72.7)		
Mucopolysaccharidosis	3 (5.7)	0 (0.0)		
Pre-treatment scheme n(%)			2.01	0.215
BU + CTX + ATG	4 (7.5)	4 (12.1)		
CTX + ATG + TBI	1 (1.9)	2 (6.1)		
FLU+CTX + ATG	48 (90.6)	27 (81.8)		
Number of infused CD34 + cells	6.19 ± 2.31	5.26 ± 1.95	1.92	0.051

### Hematopoietic reconstruction

**Fig. 1:**
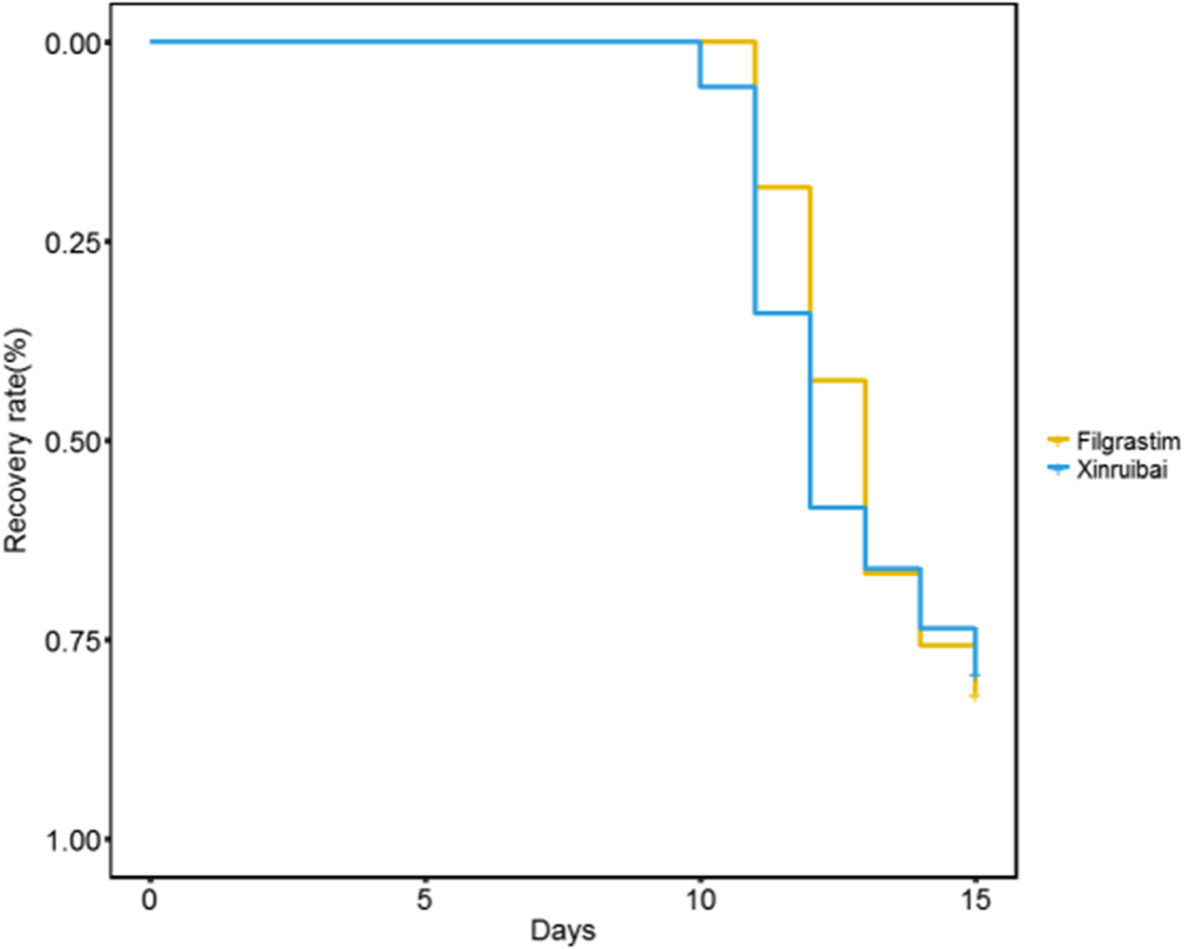
Comparison of time to neutrophil recovery between the two groups

**Fig. 2:**
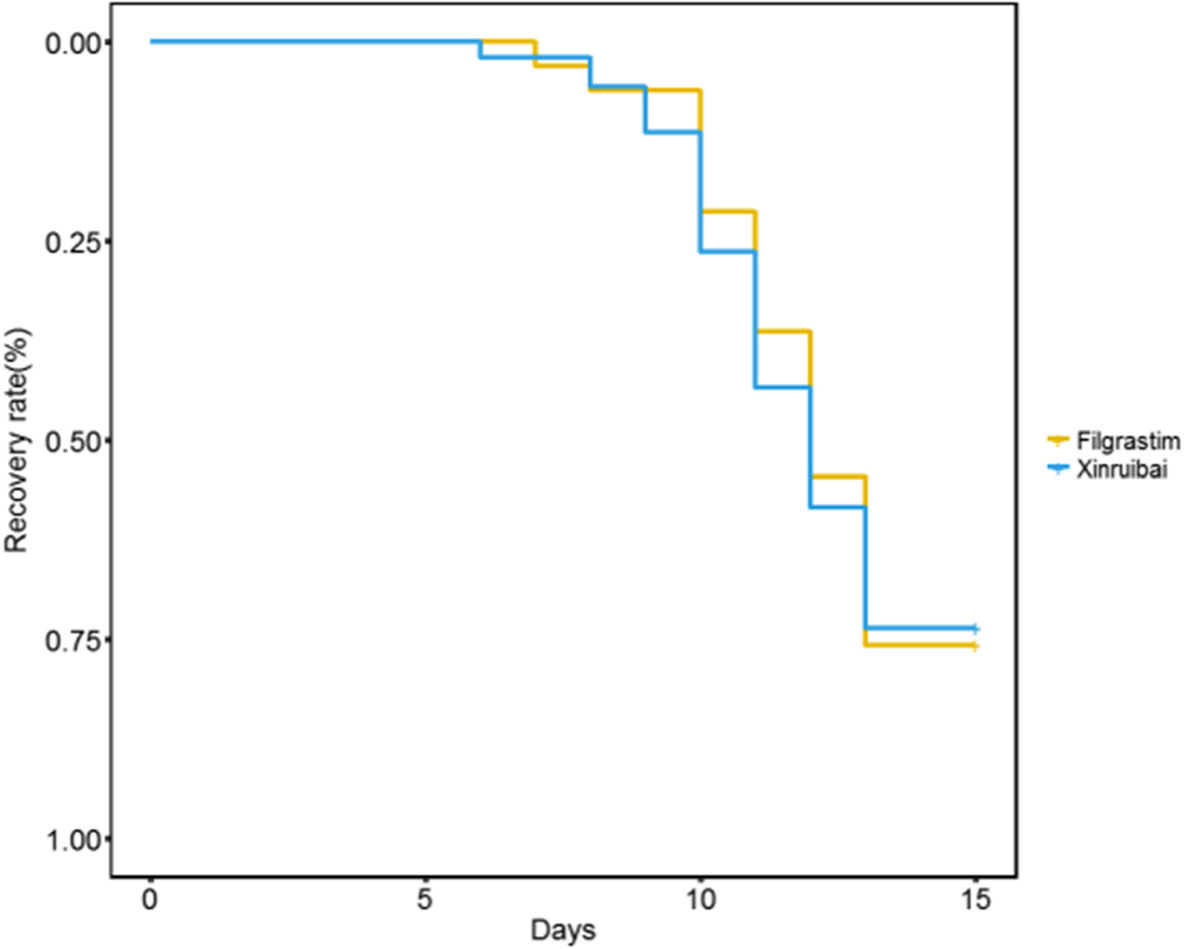
Comparison of time to platelet recovery between the two groups

The average time to neutrophil recovery was 13.42 d (SD 2.31) in the filgrastim group and 13.18 d in the xinruibai group. The average time to PLT recovery was 13.82 d (SD 5.52) in the filgrastim group and 12.92 d (SD 4.05) in the xinruibai group. The white blood cell count and neutrophil count in the filgrastim group was 2.64 G/L (SD 1.58) and 72.15% (SD 11.69), respectively, and 2.58 G/L (SD 1.48) and 75.66% (SD 9.13) in the xinruibai group, respectively; there were no significant differences between the two groups **(**Table 2**)**. A comparison of the median time to neutrophil recovery between the two groups was not significantly different (Fig. 1; *P* = 0.633). A comparison of the median time to PLT recovery between the two groups also indicated no significant difference (Fig. 2; *P* = 0.911). It seems that different conditioning regimens they used had no significant influence in the time of peripheral hematopoietic cell reconstitution.

**Table 2:** Comparison of efficacy between the xinruibai and filgrastim groups

Indicator	xinruibai group	Filgrastim group	*t value*	*P* value
Time to neutrophil recovery (d)	13.18 (2.87)	13.42 (2.31)	0.42	0.673
Time to platelet recovery (d)	12.92 (4.05)	13.82 (5.52)	0.81	0.390
White blood cell count	2.58 (1.48)	2.64 (1.58)	0.18	0.854
Neutrophil percentage	75.66 (9.31)	72.15 (11.69)	1.45	0.150

### Drug-related adverse events and complications

The common adverse events related to G-CSF include fevers, skin rashes, arthralgias, ostealgia and diarrhea. Only mild AEs were observed in the two groups, and none of the patients had AEs of grade 3 or above.

Post-transplantation complications include fevers for unknown reasons, FN, infections caused by specified pathogens, GVHD, and hemorrhagic cystitis. The two groups had no significant differences in the incidence of fever for unknown reasons and transplantation-related complications **(**Table 3**)**.

**Table 3:** Comparison of the incidence of post-transplantation complications between the two groups (case, percentage)

Indicator	xinruibai group	Filgrastim group	*X* ^*2*^	*P* value
Fever for unknown reason (UFO)				
Yes	32 (60.38)	20 (60.61)	0.0004	> 0.05
No	21 (39.62)	13 (39.39)		
Transplantation-related complications			0.8347	> 0.05
Yes	10 (18.87)	9 (37.5)		
No	43 (81.13)	24 (62.5)		

### Comparison of pain score between the two groups

**Fig. 3:**
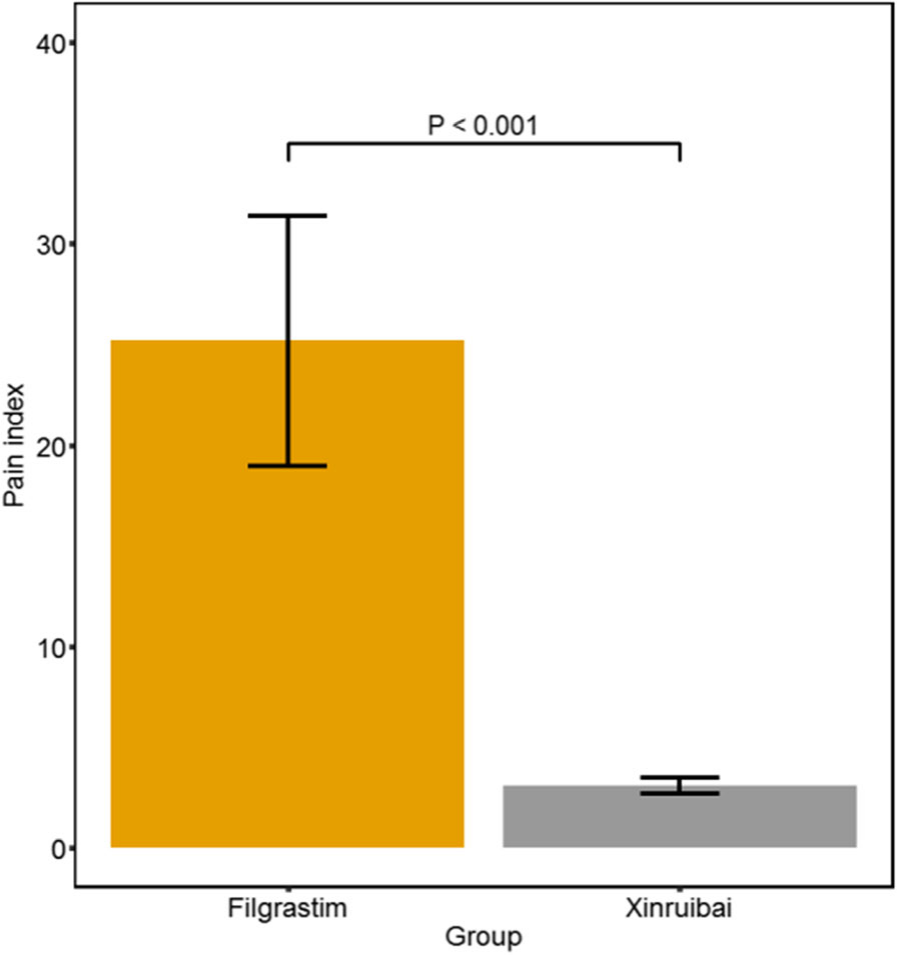
Comparison of pain index between the two groups

A single subcutaneous xinruibai injection was required, while serial filgrastim infusions were administered. Pain was scored using the modified Flacc scale for infants and the VAS scale [18–21]. Using this criterion, the pain score was 3.06 for the xinruibai group (SD 0.41) and 25.18 for the filgrastim group (SD 6.22), which indicated significant difference between the two groups (*P* < 0.001; Fig. 3).

### G-CSF and total hospitalization costs

**Fig. 4:**
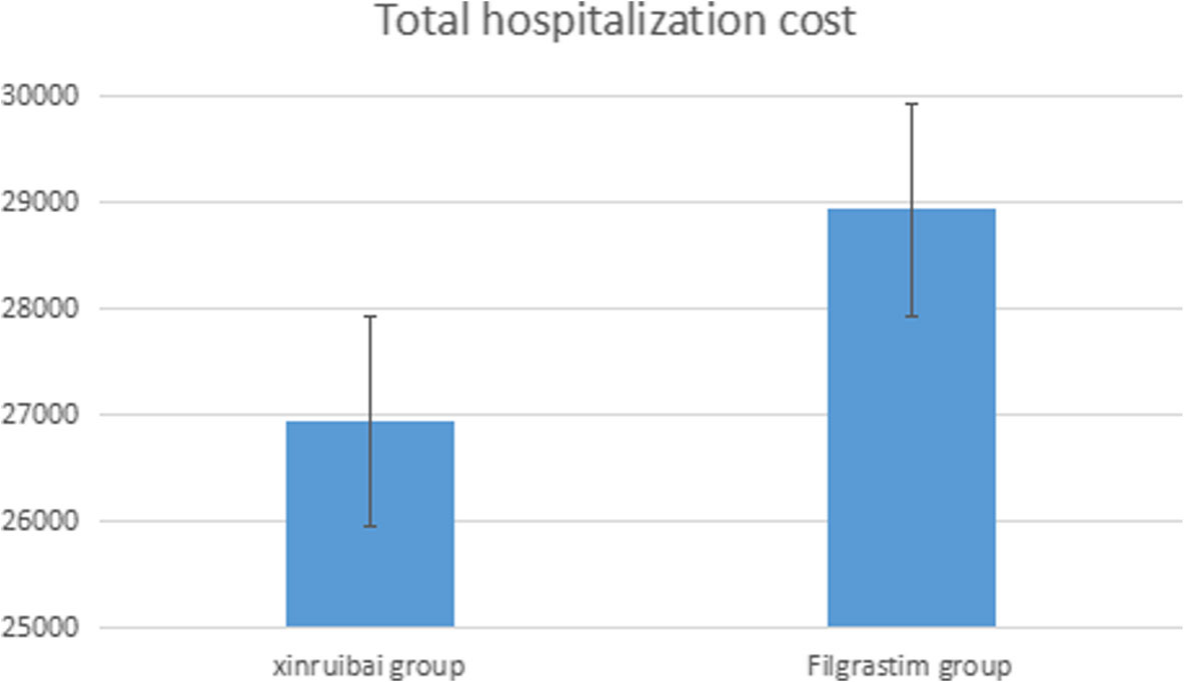
Comparison of total hospitalization costs between the two groups

The cost of one xinruibai and filgrastim injection (75μg) was 214.79 Euro and 26.27 Euro, respectively. To achieve hematopoietic reconstruction, an average of 9.78 injections (SD 2.36) of filgrastim were needed, which cost 257.11 ± 61.87 Euro. There was a significant difference compared with the xinruibai group **(**Table 4**)**. The total hospitalization cost of the xinruibai group was 26,949.77 ± 11,600.97 Euro, which was lower than the filgrastim group, but not statistically significant **(**Fig. 4**)**.

**Table 4:** Comparison of expenses between the two groups ($\overline{\mathrm{x}}$±SD, Euro)

Indicator	xinruibai group	Filgrastim group	*P* value
Expenses of GCFS injection	214.79 ± 0.00	257.11 ± 61.87	< 0.001
Total hospitalization cost	26,949.77 ± 11,600.97	28,930.52 ± 11,973.67	> 0.05

## Discussion and conclusions

The use of G-CSF after HSCT can promote hematopoietic and immune system reconstruction, while reducing the duration of FN and the incidence of infections, thereby shortening the hospital stay and lowering the total costs [14, 15]. Because of the longer half-life, only a single dose of xinruibai is needed, and the efficacy has been confirmed to be comparable to that of 10–11 injections of filgrastim during each cycle of chemotherapy [22–26]. The efficacy of xinruibai is considered comparable to that of filgrastim during chemotherapy for tumors in adults and solid tumors in children or after HSCT for lymphoma and multiple myeloma. Several guidelines have already recommended the use of xinruibai; however, as far as we know, the clinical trials on the use of xinruibai after HSCT for non-solid tumors in children are deficient.

It has been shown that the optimal duration of G-CSF is 4–7 days during chemotherapy for lymphoma [27]. According to previous studies, we performed a G-CSF infusion starting from the 5th day after HSCT. Although some studies have proposed an earlier start time for G-CSF infusions, other studies have suggested that the delayed use of G-CSF produces no apparent influence on neutrophil recovery and transplantation [28, 29]. Thus far, there have been no large-scale randomized trials indicating that xinruibai outperforms filgrastim in accelerating hematopoietic reconstruction after allo-HSCT or in shortening the time to recovery from FN after chemotherapy [9–13]; however, one meta-analysis has shown that xinruibai can more effectively reduce the incidence of FN and accelerate neutrophil recovery after allo-HSCT than filgrastim, and the former has a higher cost effectiveness [22–25, 30, 31].We conducted this retrospective study that compared the efficacy of xinruibai and filgrastim in promoting hematopoietic reconstruction after allo-HSCT for thalassemia major, aplastic anemia, leukemia, and mucopolysaccharidosis.

The efficacy of xinruibai and filgrastim was compared for different diseases under different pre-treatment regimens by analyzing the indicators of hematopoietic reconstruction and incidence of FN.

Our results indicated that compared with several injections of filgrastim, a single dose of xinruibai showed comparable efficacy in promoting hematopoietic and immune system reconstruction (neutrophil recovery) and reducing post-transplantation complications. This finding was in agreement with previous studies involving lymphoma, leukemia, and solid tumors in children [9–13]. Moreover, the total hospitalization cost in the xinruibai group was lower than the filgrastim group, but not a statistically significant difference. This finding may be due to the small sample size of our study. The cost of G-CSF was lower in the xinruibai group than the filgrastim group, which indicated that xinruibai has a higher cost effectiveness, as has been reported by Gerds et al. [31, 32].

The additional benefits of xinruibai may be associated with the up-regulation of transcription factors, such as HOXA9 and GATA3, which leads to more extensive progenitor cell differentiation and colonization [33]. It is therefore proposed that xinruibai also has an impact on the number of lymphocytes and reconstruction of the immune system after HSCT; however, no definitive conclusions have been reached given the small sample size and heterogeneity of the diseases included [34, 35].

We also paid attention to the psychological impact of several infusions of filgrastim. As analyzed above, several infusions of filgrastim increased the pain score and psychological burden for both children and their parents, thereby reducing compliance. Moreover, several infusions of filgrastim also increased the work burden for the medical staff. Xinruibai is favored in terms of cost effectiveness and convenience for the patients and medical staff.

Biases are inevitable for a retrospective cohort study, but the research has encouraging findings. Our data suggest that xinruibai and filgrastim were not significantly different in promoting hematopoietic reconstruction after allo-HSCT and preventing complications; however, xinruibai injection was more convenient, simple, effective, and safer than filgrastim. Therefore, xinruibai positions in a higher cost- effectiveness plane. But multi-center randomized controlled trials with a larger sample size are needed to confirm the findings. The long-term effect of xinruibai versus filgrastim on hematopoietic and immune system reconstruction and on the post-transplantation complications, such as GVHD are also needed to further investigation.

## Abbreviations

AEs: Adverse events
allo-HSCT: Allogeneic hematopoietic stem cell transplantation
ANC: Absolute neutrophil count
ASCO: American Society of Clinical Oncology
EORTC: Research and Treatment of Cancer
FN: Febrile neutropenia
G-CSF: Granulocyte-colony stimulating factor
GVHD: Graft-versus-host disease
Hb: Hemoglobin
PEG: Polyethylene glycol
PLT: Platelet
